# Development and Characterization of a Silver Nanoparticle-Based Hydrogel Containing Hyaluronic Acid and Allantoin for Antibacterial Burn Treatment

**DOI:** 10.3390/pharmaceutics18060724

**Published:** 2026-06-11

**Authors:** Natália Yukari Kashiwaqui, Helena Tiemi Suzukawa, Briani Gisele Bigotto, Maria Luiza Francisconi Lubanco Thomé, Danielle Lazarin Bidoia, Sueli Fumie Yamada-Ogatta, Ricardo Sérgio Couto de Almeida, Audrey Alesandra Stinghen Garcia Lonni, Mirian Sumini, Renata Katsuko Takayama Kobayashi, Gerson Nakazato

**Affiliations:** 1 Laboratory of Basic and Applied Bacteriology, Department of Microbiology, State University of Londrina, Londrina 86057-970, PR, Brazil; n.y.kashiwaqui@gmail.com (N.Y.K.); marialuiza.flubanco@gmail.com (M.L.F.L.T.); miriansumini@gmail.com (M.S.); kobayashirkt@uel.br (R.K.T.K.); 2 Laboratory of Molecular Biology of Microrganisms, Department of Microbiology, State University of Londrina, Londrina 86057-970, PR, Brazil; helena.tiemi.suzukawa@uel.br (H.T.S.); ogatta@uel.br (S.F.Y.-O.); 3 Laboratory of Mycology and Alternative Methods to Animal Use, Department of Microbiology, State University of Londrina, Londrina 86057-970, PR, Brazil; briani.gb@hotmail.com (B.G.B.); almeidar@uel.br (R.S.C.d.A.); 4 Laboratory of Electron Microscopy, State University of Maringá, Maringá 87020-900, PR, Brazil; dlbidoia2@uem.br; 5 Laboratory of Cosmeceutical Innovation and Technology, Health Sciences Center, State University of Londrina, Londrina 86057-970, PR, Brazil; audrey@uel.br

**Keywords:** allantoin, hyaluronic acid, *Acinetobacter baumannii*, resistance, burn injury

## Abstract

**Background/Objectives**: Burn injuries represent a global public health concern, accounting for approximately 265,000 deaths annually and often leading to severe infections. With the increasing prevalence of multidrug-resistant (MDR) bacteria, innovative therapeutic strategies such as nanoparticle-based topical formulations have gained attention. This study proposed the development of a hydrogel for burn treatment containing biogenic silver nanoparticles (BioAgNPs), hyaluronic acid (HA), and allantoin (AL). **Methods**: BioAgNPs were previously characterized by transmission electron microscopy (TEM) and incorporated into a hydrogel containing HA and AL, which was physicochemically characterized by pH, spreadability, and energy-dispersive X-ray spectroscopy (EDX). Antibacterial activity was evaluated by broth microdilution, agar diffusion, and time–kill assays against standard and MDR bacterial strains. Cytotoxicity was assessed using the MTT assay in L929 cells, and wound-healing potential was investigated through an in vitro scratch assay to evaluate cell migration and proliferation. **Results**: BioAgNPs exhibited antibacterial activity against reference strains and MDR isolates, determining the minimum inhibitory concentrations (MIC) and minimum bactericidal concentrations (MBC). HA and AL were non-toxic, while BioAgNPs demonstrated low cytotoxic activity. Although HA and AL did not exhibit antibacterial properties, they promoted cell migration and proliferation. The formulation exhibited physicochemical and pharmaceutical stability, showing suitable properties for topical use, and presented significant antimicrobial action, with bacterial elimination occurring within 2 h of contact, except for *S. aureus*. **Conclusions**: Thus, the hydrogel presents a promising alternative for the topical treatment of infected burns, with potential application in combating multidrug-resistant bacteria, being able to eliminate MDR *Acinetobacter baumannii*.

## 1. Introduction

Burn injuries represent a significant public health challenge and are associated with high morbidity and mortality rates, being the fourth most common type of wound worldwide, surpassed only by injuries resulting from car accidents, falls and physical assaults [1,2]. Infections are one of the most common and serious complications in burn cases, being strongly related to an increased risk of mortality among burn patients [3,4]. Furthermore, burn wounds are highly susceptible to infection, particularly by multidrug-resistant (MDR) bacteria, making the development of new antibacterial treatments to combat these strains extremely important [5,6,7]. The World Health Organization classified the bacterium *Acinetobacter baumannii* as a critical pathogen due to its multiple virulence factors and its ability to develop resistance to antibiotics [8]. Infections associated with intense immune response result in one of the most complex wounds to heal [9].

In this context, the use of hydrogels in burn treatment has proven advantageous, due to their beneficial properties for the healing process, including the ability to provide a moist and refreshing environment for burns, regulating the moisture of the wound bed and facilitating the growth of granulation tissue and wound healing [10,11]. Moreover, hydrogels are non-adherent, causing less pain and reducing the need for dressing changes. The microporous structure of hydrogels also allows for the incorporation of various active ingredients, such as antimicrobial and wound healing compounds, benefiting the healing process [11,12,13].

Silver nanoparticles can potentially be used in burn treatment due to their excellent antibacterial activity. Silver-based formulations are widely used but present limitations such as cytotoxicity and uncontrolled release [14,15]. Many dressings and formulations already use silver as an antibacterial agent, namely silver sulfadiazine, which is considered the gold standard for burn treatment [16,17,18,19]. Silver nanoparticles present increased antibacterial properties, due to their nanometric size and high surface area to volume ratio, allowing the nanoparticles to combat bacterial biofilms and multidrug-resistant strains [20,21,22,23].

The use of wound healing agents, such as hyaluronic acid and allantoin, can enhance burn healing. Hyaluronic acid composes the extracellular matrix of the dermis, promoting cell migration, proliferation, angiogenesis and tissue repair [24,25]. Allantoin is a bioactive compound with properties that promote healing, stimulate cell proliferation, and clear necrotic tissue, thus favoring the wound healing process [26,27]. Additionally, this compound promotes burn hydration and is considered biocompatible [12]. Although hydrogels containing silver nanoparticles have been investigated, few studies have combined biogenic silver nanoparticles with compounds that promote tissue regeneration, such as hyaluronic acid and allantoin [28,29,30].

Thus, the development of a hydrogel containing silver nanoparticles, hyaluronic acid, and allantoin represents a promising approach for the treatment of burns, exhibiting antibacterial activity to prevent and combat infections, including those of multidrug-resistant bacteria, and healing activity to aid in wound closure.

## 2. Materials and Methods

During the preparation of this manuscript, the authors used ChatGPT Classic (https://chatgpt.com/g/g-YyyyMT9XH-chatgpt-classic, accessed on 7 June 2026) for English language translation and grammatical refinement. Following the use of this tool, the authors reviewed and edited the content to ensure technical accuracy and take full responsibility for the final version of the manuscript.

### 2.1. Bacterial Strains

The bacteria used for the antibacterial activity tests were the standard strains *Staphylococcus aureus* ATCC 6538, *Acinetobacter baumannii* ATCC 19606, and *Pseudomonas aeruginosa* ATCC 27853, obtained from the bacterial collection of the Basic and Applied Bacteriology Laboratory of the State University of Londrina (UEL), Londrina, Paraná State, Brazil. Multidrug-resistant clinical isolates of *A. baumannii* (183, 289, 791, 846, and 860), isolated from patients at the University Hospital of Londrina (Paraná, Brazil), were kindly provided by Prof. Dr. Eliana Carolina Vespero. These clinical isolates were reviewed and approved by the, Research Ethics Committee Involving Human of the State University of Londrina project CAAE 80467624.2.000.5231, approved on 17 September 2024.

Samples were anonymized and cannot be traced back to individuals. At this moment, the bacterial isolates are located in Universitary Hospital (HU-UEL) as bacterial collection. The bacterial strains were stored at −20 °C in Brain Heart Infusion (BHI) (Difco, Franklin Lakes, NJ, USA) broth supplemented with 20% glycerol (Sigma-Aldrich, St. Louis, MO, USA).

### 2.2. Materials

Allantoin (AL; Inlab©, Madrid, Spain) and hyaluronic acid (HA; Purifarma©, Anápolis, Brazil) were commercially acquired. Biogenic silver nanoparticles (BioAgNPs) were obtained from the start-up GRAL Bioativos LTDA (NanoVerdeAg^®^, Londrina, Brazil). BioAgNPs were synthesized using *Trichilia catigua* (catuaba) extract through a patented process (BR 102021016375-5). All culture medium were obtained commercially: BHI broth (Difco©, USA), Glycerol (Sigma-Aldrich©, USA), Mueller-Hinton (MH) broth (Difco©, USA), MH agar (Difco©, USA), Dulbecco’s Modified Eagle Medium (DMEM; Gibco©, Thermo Fisher Scientific, Waltham, MA, USA), Fetal Bovine Serum (FBS; Gibco©, Thermo Fisher Scientific, USA), 3-(4,5-dimethylthiazol-2-yl)-2,5-diphenyltetrazolium bromide (MTT; Invitrogen©, Carlsbad, CA, USA), Nutrient agar (Difco©, USA).

### 2.3. Characterization of Biogenic Silver Nanoparticles

The average particle size, zeta potential, and polydispersity index (PDI) of the BioAgNPs were obtained from a Technical Report issued by the Technological Research Institute (TRI), referring to the production of pilot batches of silver nanoparticles synthesized via a biogenic route by Gral Bioativos.

#### Transmission Electron Microscopy

The size and morphology of the BioAgNPs were determined by transmission electron microscopy (TEM), using a JEOL JEM-1400 microscope (Tokyo, Japan), operating at an acceleration voltage between 40 kV and 120 kV. This analysis was performed at the Transmission Electron Microscopy Laboratory of the State University of Maringá (UEM), by Prof. Dr. Danielle Lazarin Bidóia. The samples were previously prepared on the grid, following standard protocols. The images were captured with a digital camera coupled to the TEM system.

### 2.4. Determination of the Minimum Inhibitory Concentration (MIC) and Minimum Bactericidal Concentration (MBC)

MIC and MBC values were determined using the broth microdilution assay, according to CLSI M100 standards [31]. The broth microdilution assay was performed in 96-well microplates using MH broth. BioAgNPs were tested at concentrations ranging from 2000 to 0.976 µM, HA from 8 to 0.062 mg/mL, and AL from 2.5 to 0.019 µg/mL against 5 × 10^5^ CFU/mL of each microorganism. The plates were incubated at 37 °C for 24 h, and the MIC values were defined as the lowest concentration at which no visible turbidity was observed. For MBC determination, 10 µL aliquots from each well were subcultured onto MH agar plates and incubated at 37 °C for 18–24 h.

### 2.5. Cytotoxicity Assay

The cytotoxicity assay was performed following ISO 10993-5 [32], using L929 mouse fibroblast cells (ATCC CCL-1, American Type Culture Collection, Manassas, VA, USA). Cell cultures were obtained commercially by the Adolfo Lutz Institute, São Paulo State, Brazil. Cells were exposed to BioAgNPs (250–7.812 µM), HA (8–0.062 mg/mL), and AL (400–3.125 µg/mL). A suspension of 2 × 10^5^ cells per well was seeded in 96-well plates containing DMEM supplemented with 10% FBS. Cell viability was assessed using the MTT reduction assay. The concentration required to inhibit 50% of cellular metabolic activity was defined as the cytotoxic concentration (CC_50_). The selectivity index (SI) was calculated using the equation:

$$SI=\frac{{CC}_{50}}{MIC}$$

### 2.6. Scratch Assay

The induction of cell migration and proliferation by the active ingredients were evaluated using the Scratch Assay, with modifications. L929 cells were seeded at 2 × 10^6^ cells per well and incubated for 24 h at 36 °C under 5% CO_2_. A linear scratch was created using a 200 µL Gibson-type pipette tip, and wells were washed with phosphate-buffered saline (PBS). Different concentrations of the active ingredients, diluted in DMEM supplemented with 10% FBS, were tested in their respective wells: 8, 4, 2, and 1 mg/mL of HA and 200, 100, 50, and 25 μg/mL of AL. Untreated controls received DMEM with 10% FBS only. The plate was incubated at 36 °C with a carbon dioxide tension of 5% (*v*/*v*), and images were captured every 6 h of incubation using a microscope at 4× objective. Wound areas were quantified using ImageJ software version 1.54p.

### 2.7. Binary Association of BioAgNPs with HA and AL

Binary interactions between BioAgNPs and HA or AL regarding antibacterial activity were evaluated using the checkerboard assay, with modifications.

In the 96-well plate, 50 µL of MH broth was distributed in each well. BioAgNPs were serially diluted (2000–0.49 µM), and 50 µL of HA (8 mg/mL) or AL (100 µg/mL) was added to the corresponding wells. Bacterial suspensions were adjusted to the McFarland 0.5 scale (≅1.5 × 10^8^ CFU/mL) in saline and then diluted to obtain an inoculum of approximately 10^6^ CFU/mL. In each well, 100 µL of this bacterial inoculum was added, resulting in 10^5^ CFU/mL in each well. The plate was incubated at 37 °C for 24 h and bacterial growth was assessed visually.

### 2.8. Formulation Development

The formulations were developed based on hydroxyethylcellulose, and four distinct versions were developed: (i) base without active ingredient and without preservative, (ii) base without active ingredient containing preservative (BC), (iii) active formulation without preservative (FA), and (iv) active formulation containing preservative (FAC). Due to a pending patent application, specific formulation details cannot be disclosed at this time.

The active formulation with preservative and the base without active ingredient with preservative were subjected to physicochemical characterization and preliminary stability tests, while the active formulation without preservative and the base without active ingredient without preservative were used to evaluate microbiological activity.

The formulation base was composed of Hydroxyethylcellulose, Aqua (Water), Disodium EDTA, and Glycerin. The active formulation contains all the components of the base, in addition to the active ingredients biogenic silver nanoparticles (BioAgNP), Sodium Hyaluronate (hyaluronic acid), and Allantoin. The preservative used was a combination of Methylisothiazolinone and Phenoxyethanol, commercially known as Neolone^®^ PE (Lanxess, Pittsburgh, PA, USA).

### 2.9. Characterization of the Formulation

#### 2.9.1. Pharmaceutical Characterization

The formulations were evaluated according to the tests recommended in the Brazilian Pharmacopoeia. The tests were performed in triplicate for each formulation, and the averages were calculated.

##### Pre-Stability Test

5 mL aliquots of the samples were placed in a graduated, conical plastic test tube. The samples were centrifuged at 2800 rpm for 30 min at room temperature to detect visible changes or instabilities such as phase separation. Observation occurred at two times: macroscopic analysis after 24 h at rest and after the centrifugation test.

##### Organoleptic Tests

The appearance, color, and odor of the formulations were evaluated. Regarding appearance, the samples were placed in watch glasses and placed on a dark background. It was visually observed whether the sample under study maintained the macroscopic characteristics of the reference sample and whether alterations occurred, such as phase separation, precipitation, and turbidity.

The color analysis of the formulations was performed by visual comparison, under white light conditions, of the sample color with the color of the standard, stored under the same conditions and packaging as the sample. The visual analysis of the color of the samples was performed with 1.5 mL of the samples placed in watch glasses, placed on a dark background. Then, it was photographed and its results compared. The samples were classified by color as: normal, unaltered; slightly modified; modified; intensely modified, in both methodologies.

The odor of the samples was compared with the odor of the reference formula (kept under refrigeration), directly through smell. The sample was classified as normal, unaltered; slightly modified; modified; intensely modified.

##### Physicochemical Tests

The pH of each formulation was determined using a digital pH meter at room temperature (25 ± 5 °C), calibrated with buffer solution (pH 4.0 and 7.0). For both determinations, the electrode was inserted directly into the sample.

Density was evaluated using a glass pycnometer with a capacity of 10 mL and a monitored temperature of 20 °C. The ratio between the mass of the sample and the mass of water represents the specific density of the sample tested.

#### 2.9.2. Spreadability

The spreadability assessment consisted of using glass plates under millimeter paper to determine the surface area covered by 1 g of the sample by measuring the perpendicular diameters and calculating the average diameter, at a temperature of 25 ± 2 °C [33]. This method was repeated using pre-determined weights (2, 5, and 10 g) at 1 min intervals. 

The spreadability values as a function of the added weights were determined in triplicate, and the average was calculated.

#### 2.9.3. Preliminary Stability Assessment

The preliminary stability assessment was performed in the initial phase of product development. Over 15 days, the samples were subjected to stress conditions aimed at accelerating the emergence of possible signs of instability. They were subjected to heating in ovens for 24 h at 40 ± 2 °C and relative humidity 75 ± 5% and cooling in refrigerators for 24 h at 2 ± 2 °C, for 15 days, alternating cooling and heating cycles.

#### 2.9.4. X-Ray Diffraction Spectroscopy of the Formulation

The characterization of the lyophilized hydrogel surface and confirmation of the presence of silver were performed using X-ray diffraction spectroscopy (XDS), following the methodology of Alcântara et al. (2020) [34], using the FESEM JMS-6701F scanning electron microscope, JEOL, coupled to an energy-dispersive X-ray spectrometer, carried out by Prof. Dr. Renan da Silva Nunes and Prof. Dr. Amedea Barozzi Seabra.

### 2.10. Antibacterial Activity of the Formulation

#### 2.10.1. Agar Drop-Diffusion Assay

The antibacterial activity of the active formulation was assessed by the agar drop diffusion assay against the standard strains *Acinetobacter baumannii* ATCC 19606, *Pseudomonas aeruginosa* ATCC 27853, and *Staphylococcus aureus* ATCC 6538, and against multidrug-resistant isolates of *Acinetobacter baumannii* numbered 183, 289, 791, 846, and 860.

A bacterial suspension of 1.5 × 10^8^ CFU/mL (0.5 McFarland scale) of each tested bacterium was prepared and spread homogeneously on the surface of a nutrient agar plate using a swab. Approximately 10 µL of the active formulation and the base were deposited over the bacterial mat. The plate was incubated at 37 °C for 24 h.

#### 2.10.2. Antimicrobial Efficacy Test

The Antimicrobial Efficacy Test is a standardized test described by the Brazilian Pharmacopoeia, which consists of the intentional contamination of products with microorganisms, determining the surviving load, evaluated by colony counts [35]. This test aims to assess whether the formulation remains safe and unchanged in the face of a high microbial load for a certain period. This test was performed with modifications against standard strains *Acinetobacter baumannii* ATCC 19606, *Pseudomonas aeruginosa* ATCC 27853 and *Staphylococcus aureus* ATCC 6538 and against multidrug-resistant isolates of *Acinetobacter baumannii* numbered 183, 289, 791, 846 and 860.

Formulations (1.5 g) were inoculated with 100 bacterial µL suspensions of 1.5 × 10^8^ CFU/mL (0.5 McFarland Scale) and sampled at 0, 2, 4, 8, and 12 h. After the incubation periods, dilutions up to −4 were prepared from each formulation and 10 µL were plated in triplicate onto nutrient agar plates. The plates were incubated at 37 °C for 24 h for subsequent colony counting, the values of which were used to construct base-10 logarithmic graphs.

#### 2.10.3. Time–Kill Curve Assay

The bacterial growth and death curve was performed following the standards described in document M26-A, National Committee for Clinical Laboratory Standards, against the standard strains *Acinetobacter baumannii* ATCC 19606, *Pseudomonas aeruginosa* ATCC 27853, and *Staphylococcus aureus* ATCC 6538, and against a multidrug-resistant isolate of *Acinetobacter baumannii* 846 [36].

An active formulation with 500 µM of BioAgNP was diluted to the final concentration of 125 µM. Three microtubes were prepared for each bacterium and time point: one with the active formulation, another with the base, and the third with only culture medium to serve as a positive control. Bacterial inoculum and MH broth were added to each microtube, resulting in a final concentration of 125 µM of BioAgNPs present in the active formulation and 10^6^ CFU/mL of the bacterial inoculum. The microtubes were incubated at 37 °C, and the contents were diluted in saline (−1 to −4) and plated on MH agar for different incubation times: 0, 2, 4, 8, and 12 h. The plates were incubated overnight at 37 °C for analysis of the antibacterial activity of the BioAgNPs by counting the number of colonies surviving the treatments and constructing a log10 bacterial growth and death curve.

## 3. Results

### 3.1. Characterization of Biogenic Silver Nanoparticles

According to the Technical Report, the biogenic silver nanoparticles (BioAgNP) presented an average size of 82.73 nm, confirming that the particles fall within the nanometric range (1–100 nm) [37]. The polydispersity index (PDI) of the nanoparticles was 0.17, indicating a narrow size distribution, and the zeta potential was −23.27 mV.

**Figure 1:**
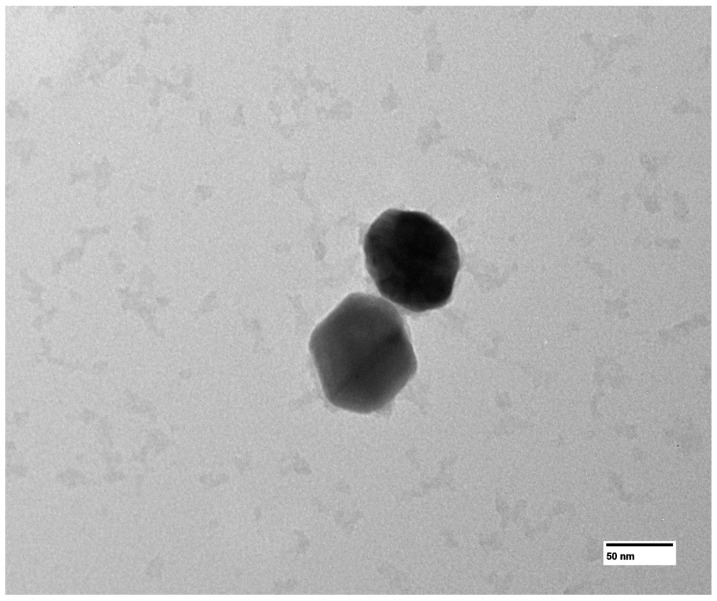
TEM micrograph of biogenic silver nanoparticles (BioAgNP).

Transmission Electron Microscopy (TEM) was performed to further evaluate the morphology and size of the BioAgNPs. The micrograph obtained from the TEM is shown in Figure 1.

As observed in Figure 1, the BioAgNPs exhibit sizes greater than 50 nm, which is compatible with the average size of 82.73 nm obtained by DLS. Image analysis of an individual nanoparticle using ImageJ software, measured at different angles due to its non-spherical morphology, yielded particle dimensions of 75.976, 90.261, 78.581, and 78.251 nm, resulting in an average size of 80.767 nm, a value very close to the DLS-derived average size of 82.73 nm. Additionally, the TEM images suggest the presence of a surface coating surrounding the nanoparticles.

### 3.2. Antibacterial Activity

Neither hyaluronic acid (HA) nor allantoin (AL) showed antibacterial activity at the tested concentrations, preventing the determination of the minimum inhibitory concentration (MIC) and minimum bactericidal concentration (MBC) values against the standard strains of *Acinetobacter baumannii*, *Pseudomonas aeruginosa*, and *Staphylococcus aureus*. Consequently, the antibacterial activity of these compounds was not further evaluated against multidrug-resistant strains.

BioAgNPs presented antibacterial activity against the three standard strains tested. The MIC and MBC values are presented in Table 1.

The MIC and MBC values obtained from the broth microdilution assay against the five *A. baumannii* isolates are presented in Table 2.

### 3.3. Cytotoxicity Assay

**Figure 2:**
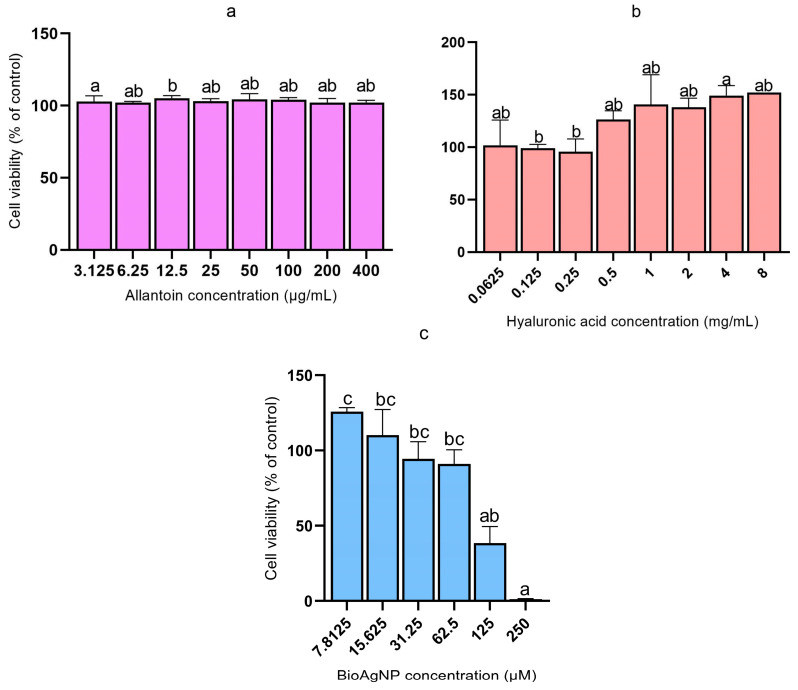
Cytotoxic activity of allantoin (**a**), hyaluronic acid (**b**) and biogenic silver nanoparticles (BioAgNP) (**c**) on L929 cells determined by MTT assay. The experiments were performed in triplicate. The letters above the columns indicate which treatments showed a significant difference.

The cytotoxicity of AL, HA, and BioAgNPs was evaluated by cytotoxicity assay and the results obtained were used to construct graphs in Figure 2, which illustrate the cytotoxic effects of each active ingredient.

Neither HA nor AL exhibited cytotoxic activity at any of the concentrations tested, making it impossible to calculate the CC50. Meanwhile, BioAgNPs exhibited low cytotoxic activity, obtaining a CC50 value of 14.239 µg/mL. From the CC50, it was possible to calculate the selectivity index (SI) of the bioAgNPs, presented in Table 3.

### 3.4. Scratch Assay

After confirming that AL and HA do not exhibit cytotoxic activity at the evaluated concentrations, the Scratch or wound closure assay was performed, which evaluated the cell migration and proliferation of L929 fibroblasts stimulated by AL and HA [38]. The Scratch assay was used as an in vitro model for preliminary evaluation of the healing potential, as it is a method widely used in the literature to study keratinocyte migration and processes related to epithelial regeneration [39,40,41].

**Figure 3:**
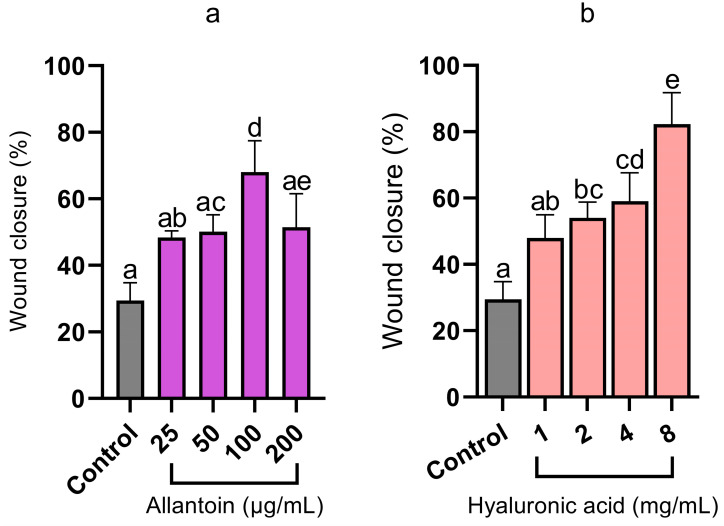
Reduction in the wound area in the L929 fibroblast monolayer induced by allantoin (AL) (**a**) and hyaluronic acid (HA) (**b**).

**Figure 4:**
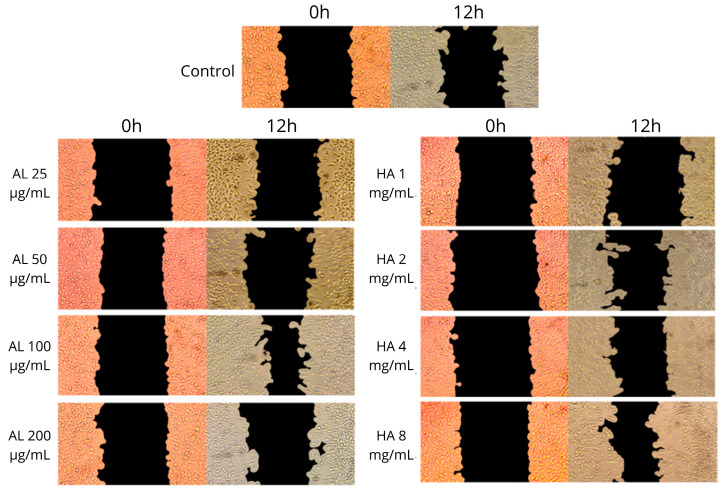
Comparison of wound closure in control cells and cells treated with different concentrations of allantoin (AL) and hyaluronic acid (HA) in the scratch assay after 12 h. AL: allantoin; HA: hyaluronic acid. Prior to analysis, all images (including Figure 4) were cropped to identical dimensions and contrast was adjusted to improve visualization of the wound edges. Wound closure was quantified using the Wound Healing Size Tool plugin in ImageJ software (National Institutes of Health, Bethesda, MD, USA). To facilitate automated wound detection by the plugin, the cell-free region was manually highlighted in black, allowing accurate segmentation and area measurement. Magnification of 40×.

The result of the Scratch assay after 12 h of treatment is presented in Figure 3 and Figure 4.

Figure 3 shows the percentage of wound closure in the control group and in cells treated with AL and HA at different concentrations. The control group exhibited little migration and proliferation of cells compared to the cells treated with AL and HA, especially at concentrations of 100 µg/mL and 8 mg/mL, respectively. The letters above the columns indicate which treatments showed a significant difference, with HA at 8 mg/mL showing a significant difference compared to the other treatments and the control group, resulting in greater wound closure. Although AL at 100 µg/mL did not show a significant difference compared to AL at 25, 50, and 200 µg/mL and HA at 1, 2, and 4 mg/mL, the result for this concentration was superior to the others, being surpassed only by HA at 8 mg/mL.

### 3.5. Binary Association of BioAgNPs with HA and AL

When multiple active ingredients are incorporated into the same formulation, interaction between them may occur, which can modify their biological activity. Regarding antimicrobials, it has been reported that AgNPs combined with tetracycline or cefixime lose antibacterial activity against *P. aeruginosa*, although each compound is effective when used individually [42]. Therefore, the association of BioAgNPs with HA and AL was evaluated to verify if the combination with HA or AL affects the MIC of BioAgNPs. The results of the assay are presented in Table 4.

Compared with BioAgNP alone, the association with HA resulted in an increased MIC for *A. baumannii*, while the association of BioAgNPs with AL at 100 µg/mL resulted in higher MICs for both *A. baumannii* and *S. aureus*. Although the MIC increased in some combinations, the highest value observed was 125 µM or 13,483 µg/mL, which remains lower than the CC_50_ of BioAgNPs (132.071 µM or 14.239 µg/mL).

### 3.6. Formulation Characterization

The hydrogel formulation was based on hydroxyethylcellulose (HEC), a water-soluble polymer derived from cellulose. BioAgNPs were incorporated at a concentration of 125 µM (13.483 µg/mL), based on the results of the association assay. Hyaluronic acid (HA) and allantoin (AL) were incorporated at 8 mg/mL and 100 µg/mL, respectively.

**Figure 5:**
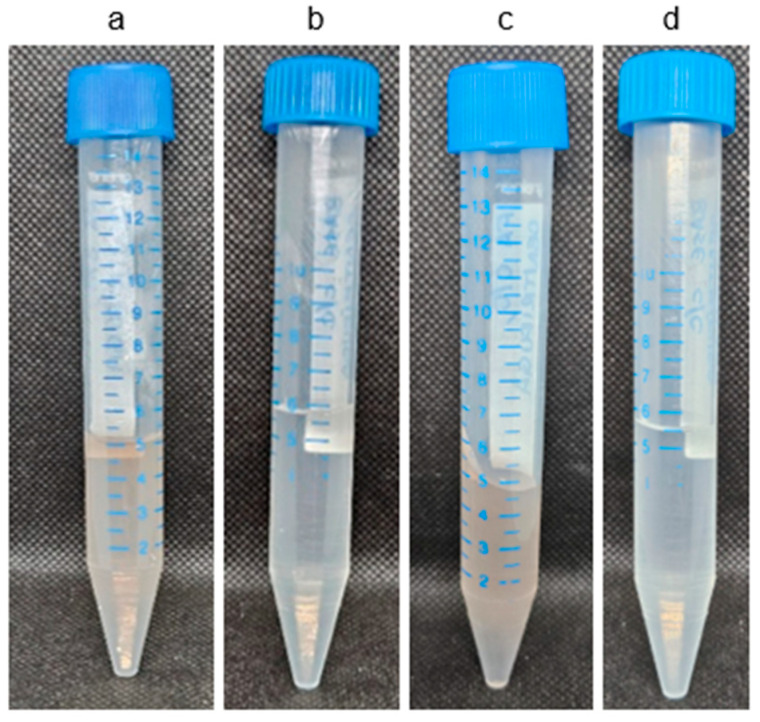
Active formulation containing preservative (FAC) (**a**) and base formulation without active ingredients and preservative (BC) (**b**) before the centrifugation test, and FAC (**c**) and BC (**d**) after centrifugation.

The first pharmaceutical characterization assay performed was the centrifugation test to assess pre-stability immediately after sample preparation. Images of the formulation before and after centrifugation are presented in Figure 5.

Figure 5 presents the base without active ingredients and with preservative (BC) and the formulation with active ingredients and preservative (FAC) before and after pre-stability centrifugation test. No phase separation, precipitation of active ingredients, or turbidity of the samples was observed indicating satisfactory initial stability and suitability for subsequent analyses.

**Figure 6:**
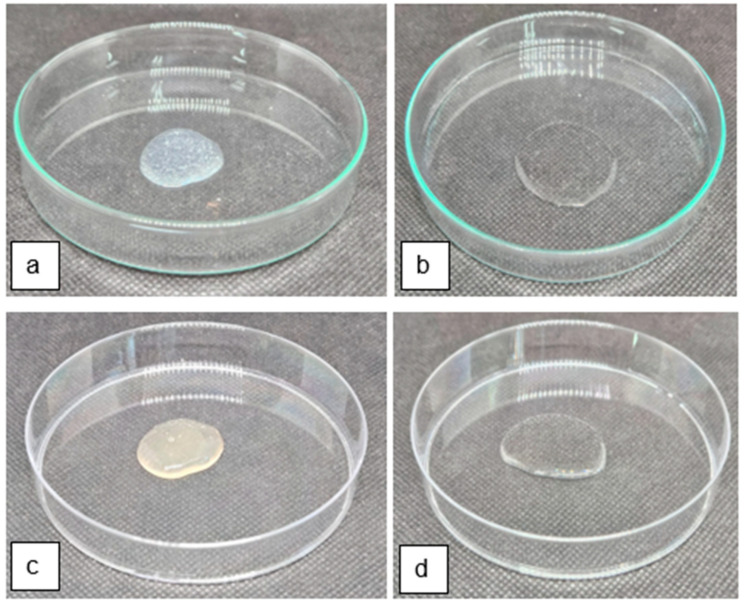
Organoleptic evaluation of the active formulation containing preservative (FAC) (**a**) and the base formulation without active ingredients and with preservative (BC) (**b**) before the preliminary stability study, and FAC (**c**) and BC (**d**) after exposure to alternating temperature cycles (4 °C/40 °C) for 15 days.

Following this test, BC and FAC were subjected to a preliminary stability study, consisting of temperature variation cycles over 15 days. Organoleptic, physicochemical, and spreadability parameters were evaluated before and after the test. Images are presented in Figure 6.

Regarding appearance, BC presented a slightly viscous consistency and a transparent color. FAC, on the other hand, exhibited transparent, slightly brown coloration and the presence of small air bubbles. No precipitation, turbidity, or phase separation was observed in either formulation.

Among the physicochemical evaluations, the pH and relative density of the formulations were determined. The pHs of BC and FAC before and after the preliminary stability test were the same at pH = 5, indicating that there was no change in this parameter. The relative density is presented in Table 5.

Regarding odor, no change was observed between FAC and BC, both maintaining a normal and unchanged scent.

**Figure 7:**
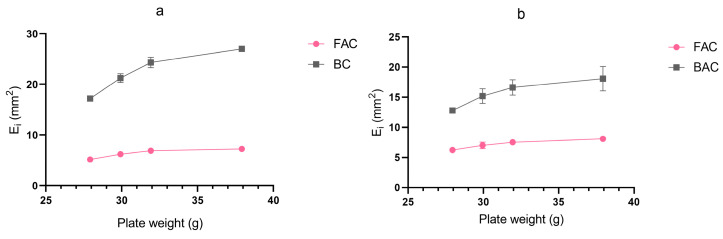
Spreadability graphs of the formulation with active ingredients and preservative (FAC) and of the base without active ingredients and with preservative (BC) before and after the preliminary stability test. (**a**) Spreadability of FAC and BC before the preliminary stability test; (**b**) Spreadability of FAC and BC after the preliminary stability test. Ei: area occupied by the sample after weight application.

The spreadability assessment demonstrated that BC, which exhibited lower viscosity than FAC, spread over a larger surface area under applied force. In contrast, FAC showed reduced spreadability, attributable to the higher viscosity imparted by hydroxyethylcellulose and hyaluronic acid. The spreadability values of FAC and BC before and after the preliminary stability test are shown in Figure 7.

**Figure 8:**
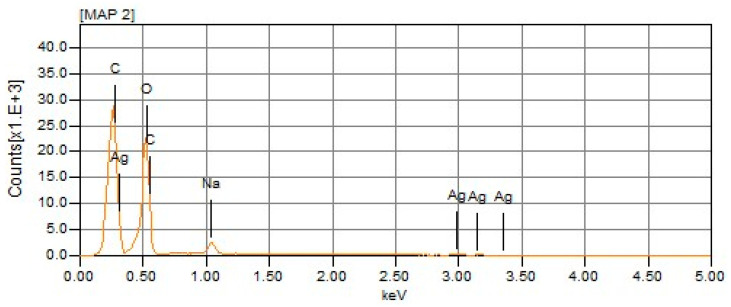
Analysis of the elemental composition of the active formulation by energy-dispersive X-ray spectroscopy.

In addition to the pharmaceutical characterization assays, energy-dispersive X-ray spectroscopy (EDS) was performed to determine the elemental composition of the formulation [43]. The EDS result is presented in Figure 8.

### 3.7. Antibacterial Activity of the Formulation

#### 3.7.1. Agar Drop-Diffusion Assay

**Figure 9:**
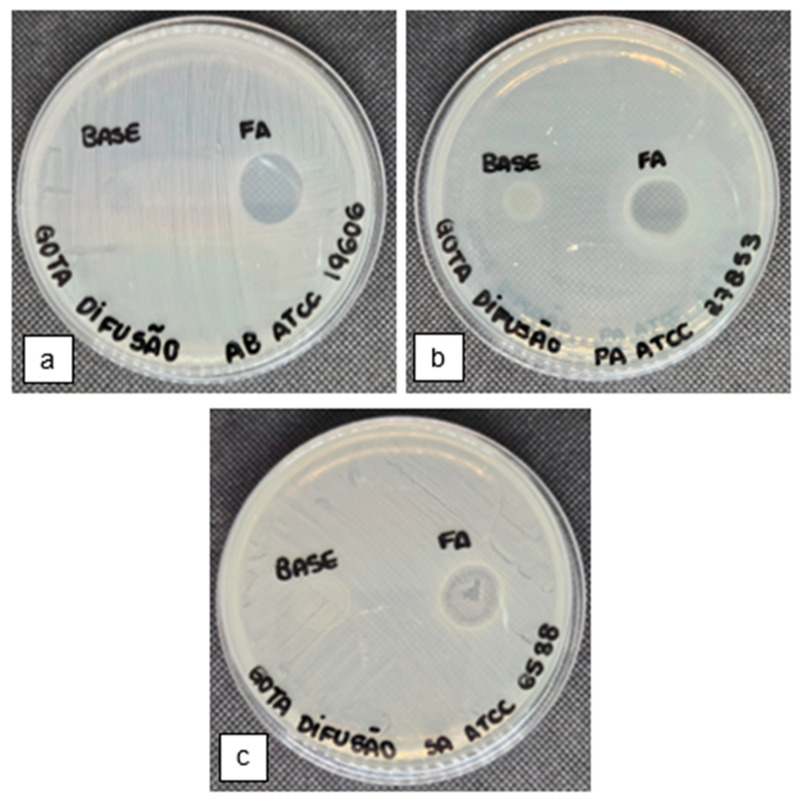
Agar drop-diffusion assay of the active formulation (FA) and the base formulation against standard strains of *Acinetobacter baumannii* ATCC 19606 (**a**), *Pseudomonas aeruginosa* ATCC 27853 (**b**), and *Staphylococcus aureus* ATCC 6538 (**c**). AB: *Acinetobacter baumannii*; FA: active formulation; PA: *Pseudomonas aeruginosa*; SA: *Staphylococcus aureus*.

The agar drop-diffusion assay was performed as a preliminary test of the antibacterial activity of FA and base formulation. The results of the agar drop diffusion assay are shown in Figure 9.

#### 3.7.2. Antimicrobial Efficacy Test

**Figure 10:**
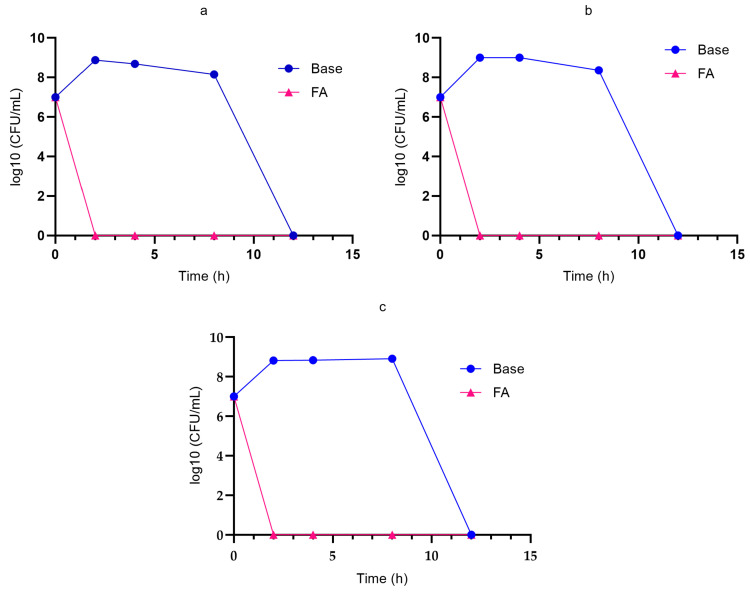
Results of the bacterial efficacy test of the active formulation (FA) and the base formulation against *Acinetobacter baumannii* ATCC 19606 (**a**), *Pseudomonas aeruginosa* ATCC 27853 (**b**) and *Staphylococcus aureus* ATCC 6538 (**c**). CFU: colony-forming units.

The antimicrobial efficacy test is an assay recommended by the Brazilian Pharmacopoeia to evaluate the effectiveness of preservatives in pharmaceutical formulations. In this study, the test was adapted to assess the antibacterial activity of BioAgNPs, the active antimicrobial component of the formulation, against bacterial contamination within the formulation itself. The bacteria were inoculated directly into aliquots of the FA. The results of the antimicrobial efficacy test are presented in Figure 10.

In all three graphs shown in Figure 10, it is possible to observe that, in the base without active ingredients, bacterial viability was maintained for up to 12 h. The decline observed in the control curves is not attributable to antibacterial activity, but rather to nutrient depletion, since no culture medium was added during the assay. In contrast, FA completely eliminated contamination by *A. baumannii*, *P. aeruginosa*, and *S. aureus* within 2 h of contact, demonstrating strong antibacterial activity.

**Figure 11:**
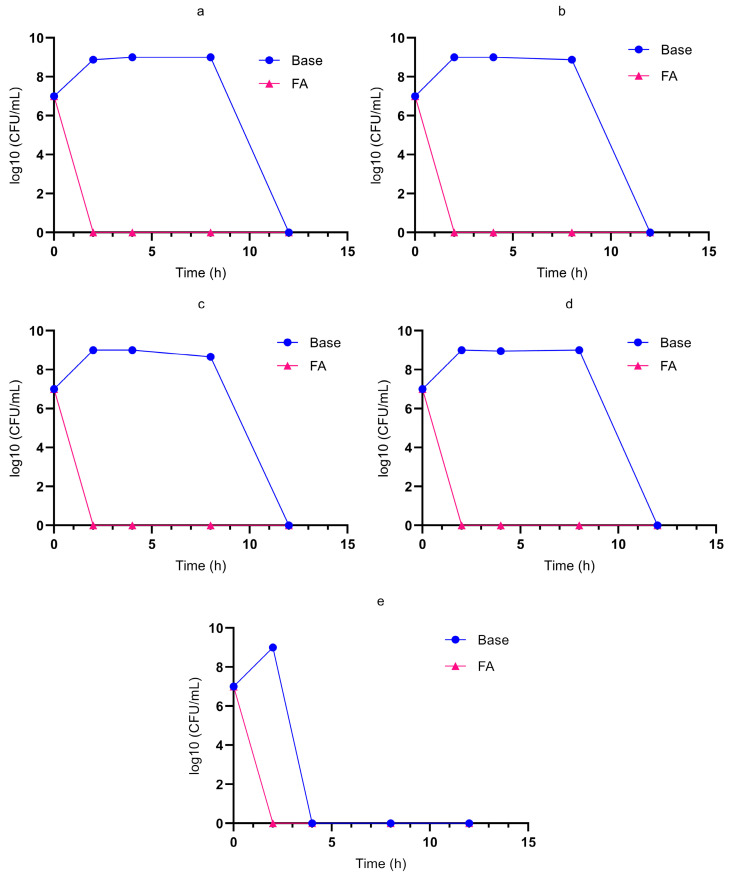
Results of the antimicrobial efficacy test of the active formulation (FA) and the base formulation against multidrug-resistant isolates of *Acinetobacter baumannii* 183 (**a**), 289 (**b**), 791 (**c**), 846 (**d**) and 860 (**e**). CFU: colony-forming units.

This test was also performed against multidrug-resistant isolates of *A. baumannii*, the results of which are shown in Figure 11.

Similarly to the reference strains, the multidrug-resistant *A. baumannii* isolates were completely eliminated from the FA within 2 h of contact, demonstrating the strong antibacterial activity of the formulation, while the base showed no activity against the bacteria.

#### 3.7.3. Time–Kill Curve Assay

**Figure 12:**
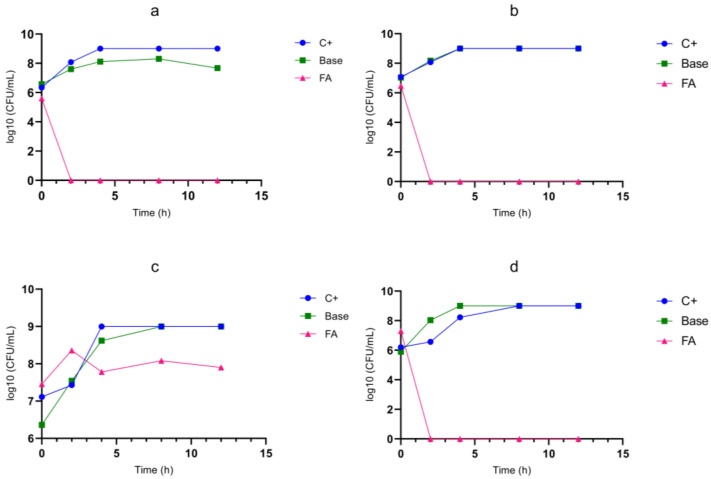
Bacterial growth and death curve of *Acinetobacter baumannii* ATCC 19606 (**a**), *Pseudomonas aeruginosa* ATCC 27853 (**b**), *Staphylococcus aureus* ATCC 6538 (**c**) and multidrug-resistant *Acinetobacter baumannii* isolate 846 (**d**) against the active formulation (FA) and the base. C+: positive control; FA: active formulation; CFU: colony-forming units.

Finally, the time–kill curve assay was performed, which is similar to the antimicrobial efficacy test, but with the addition of culture medium, favoring the bacterial growth. The bacteria evaluated in this test were the three standard strains and isolate 846, selected as a representative of the multidrug-resistant isolates since it is the only pan-resistant strain. The results are presented in Figure 12.

Figure 12c shows the growth curve of *S. aureus*, which differs from that observed in the antimicrobial efficacy test, with the bacteria being able to survive even after 12 h in the FA. The growth and death curves of *P. aeruginosa* ATCC 27853, *A. baumannii* ATCC 19606, and isolate 846, when treated with FA, exhibited an exponential decrease.

The time–kill curves of *A. baumannii* ATCC 19606 and isolate 846 shown in Figure 12a,d were similar to the curves of the antimicrobial efficacy test, with complete bacterial elimination within 2 h of contact with FA, while the positive control and the base supported bacterial growth, due to the presence of culture medium.

## 4. Discussion

### 4.1. Characterization of Biogenic Silver Nanoparticles

The obtained PDI value (0.17) indicates a narrow and homogeneous size distribution, since values between 0.1 and 0.25 are associated with monodisperse nanoparticle populations. This suggests that the biogenic synthesis process produced nanoparticles with relatively uniform sizes.

The zeta potential indicates the surface charge of the nanoparticles and is commonly used as an indicator of colloidal stability, as it reflects the tendency of particles to repel or aggregate. Stability can be assessed electrostatically, such that if a nanoparticle solution presents a homogeneity of charges, whether all negative or all positive, the tendency is for repulsion to occur and for them to remain as nanoparticles [44]. BioAgNPs exhibited a zeta potential of −23.27 mV, which indicates electrostatic stability, since values greater than +20 mV or lower than −20 mV are generally considered stable dispersions [45].

The TEM observations corroborated the particle size obtained by DLS, confirming that the nanoparticles are within the nanometric range. Additionally, the images suggest the presence of a surface coating surrounding the nanoparticles, a common feature in biologically synthesized nanomaterials. In green synthesis processes, biological extracts may act as both reducing and stabilizing agents, forming a coating around the nanoparticles [46,47].

This surface coating may contribute to the stability of the nanoparticles by increasing electrostatic repulsion and reducing particle aggregation. Furthermore, depending on its composition, the coating may also reduce the potential toxicity of nanoparticles in biological systems [46]. The biomolecules present in the extract can adsorb onto the nanoparticle surface through electrostatic and Van der Waals interactions during synthesis, forming this stabilizing layer [48].

### 4.2. Antibacterial Activity

Shukla, Srivastava and Mishra (2025) reported antibacterial activity of HA against *Escherichia coli* and *Staphylococcus aureus* using the agar well diffusion assay at concentrations ranging from 0.5 to 3.5 g/L [49]. However, previous studies demonstrated that high molecular weight HA shows better results in reducing bacterial contamination [50,51]. The antibacterial activity of HA is attributed to the presence of hydroxyl and acid groups, so the larger the HA molecule, the better its activity (Shukla; Srivastava; Mishra, 2025 [49]). In the present study, the HA used had a low molecular weight (<1000 kDa), which likely explains the absence of detectable antibacterial activity under the experimental conditions.

Regarding the antibacterial activity of AL, Lakshmanan et al. (2019) [52] reported MIC values of 4 μg/mL against Bacillus subtilis and 8 μg/mL against *S. aureus*, *E. coli*, and *Klebsiella pneumoniae*. This discrepancy between those findings and the results of the present study may be related to the origin of AL, since Lakshmanan et al. (2019) obtained the compound from the plant *Cleome viscosa* L., which may contain structurally distinct forms or residual bioactive compounds that enhance antibacterial activity [52]. In the present work, both HA and AL were incorporated into the formulation primarily as wound-healing agents; therefore, the absence of intrinsic antibacterial activity does not compromise the objectives of this study.

In Table 1, it is possible to observe that the MIC and MBC values obtained were lower for *A. baumannii* and *P. aeruginosa* when compared to *S. aureus*. Since both *A. baumannii* and *P. aeruginosa* are Gram-negative bacteria, these results suggest that the BioAgNPs exhibit a better antibacterial action against this group than against Gram-positive bacteria.

This may be attributed to the presence of an outer membrane in the cell wall of Gram-negative bacteria. One of the proposed mechanisms of action of AgNPs is adhesion to the bacterial surface, leading to nanoparticle accumulation, which results in membrane destabilization and, consequently, increased permeability. The outer membrane of Gram-negative bacteria contains proteins that can bind AgNPs, forming complexes with oxygen, phosphorus, nitrogen, or sulfur, which results in the inactivation of these proteins. Furthermore, Gram-negative bacteria have water channels called porins that facilitate the entry of nanoparticles, enhancing antibacterial activity [53].

Gram-positive bacteria, on the other hand, do not present an outer membrane; therefore, there are no porins to facilitate the entry of AgNPs, nor proteins for adhesion, but rather a thick layer of peptidoglycan, which makes it difficult for nanoparticles to penetrate [53]. These differences in the two bacterial types may be related to the enhanced antibacterial activity exhibited by BioAgNPs against Gram-negative bacteria compared with Gram-positive bacteria.

The sixth column of Table 1 shows the MBC/MIC ratio, which is commonly used to determine whether an antimicrobial agent exhibits bactericidal or bacteriostatic activity in vitro. If the MBC/MIC ratio is less than or equal to 4, the action is bactericidal; if it is greater than 4, it is bacteriostatic [54]. Thus, based on this criterion, it is possible to conclude that BioAgNPs exhibit bactericidal action against *P. aeruginosa* and bacteriostatic action against *S. aureus* and *A. baumannii*.

In the study of Tian et al. (2022), AgNPs with an average diameter of 18.52 nm and a zeta potential of −44.72 mV, synthesized using tannic acid and sodium alginate, exhibited a MIC of 31.25 μg/mL for *S. aureus* [55]. This MIC value was considerably higher than the one obtained in the present study (6.742 μg/mL), demonstrating that, despite being the same bacterial species and a similar antimicrobial, substantial variations in MIC values may occur. The difference may be attributed to the negative zeta potential of −44.72 mV, which is more negative than the zeta potential of BioAgNP (−23.27 mV), since the surface charge influences the electrostatic adhesion of AgNP to the bacterial surface, which has a negative charge. Thus, more positive nanoparticles tend to adhere more to the bacteria, destabilizing the bacterial cell wall [21].

Villani et al. (2025) synthesized AgNPs with an average size of 24 nm and a zeta potential of −15 mV using *Eucalyptus globulus* extract and obtained a MIC of 20 μM against *P. aeruginosa*, which is close to the MIC presented in Table 1 [56]. The same was observed when compared with the MIC values obtained by Michailidu et al. (2025), who evaluated 2 to 22 nm AgNPs synthesized from *Cannabis sativa* extract and observed MICs from 0.6 to 1.1 μg/mL for different *P. aeruginosa* strains, which are close to 0.843 to 1.685 μg/mL [57]. In the article by Manga et al. (2024), the 50.29 nm AgNPs, synthesized from *Cymbopogon citratus* extract, exhibited a MIC of 2 μg/mL for three *P. aeruginosa* strains tested [58]. Thus, it is possible to observe that the result obtained in the present study was compatible with the literature.

Wypij et al. (2021) [59] reported MIC and MBC values of 128 and 256 μg/mL against *S. aureus* and 8 and 64 μg/mL against *P. aeruginosa*, respectively. The MIC and MBC values obtained in the present study were lower than those obtained by Wypij et al. (2021) [59], indicating excellent results for the antibacterial activity of the BioAgNPs. Wypij et al. (2021) [59] also observed that the antibacterial activity of AgNPs is more efficient against Gram-negative bacteria than against Gram-positive bacteria, showing a higher MIC for *S. aureus* than for *P. aeruginosa*, *E. coli*, and *K. pneumoniae*.

Khojasteh-Taheri et al. (2023) synthesized AgNPs (14.8 nm and −3.4 mV) from *Caccinia macranthera* extract, which were tested against *S. aureus*, *P. aeruginosa*, and *A. baumannii*, obtaining MICs of 31.25 μg/mL, 62.5 μg/mL, and 62.5 μg/mL, respectively [60]. The MBCs for *S. aureus*, *P. aeruginosa*, and *A. baumannii* were 250 μg/mL, 500 μg/mL, and 31.25 μg/mL, respectively. In this case, it is possible that, due to the low zeta potential value, the AgNPs may exhibit low electrostatic repulsion, tending to agglomerate and form larger particles [21]. Notably, all MIC and MBC values reported by Khojasteh-Taheri et al. (2023) were higher than those observed in the present study (Table 1), reinforcing the strong antibacterial activity of BioAgNPs even at low concentrations [60].

For *A. baumannii*, Abootalebi et al. (2021) reported that 10 nm AgNPs synthesized from *Ferula asafoetida* extract exhibited a MIC of 2 μg/mL, a considerably low value [61].

Differences in antibacterial activity may be attributed to several factors, including the extract used during synthesis, nanoparticle size, and zeta potential [21,53,62]. In the green synthesis, biomolecules present in the extract form a coating on the nanoparticles’ surface, keeping them stabilized. The components of the coating can facilitate the adhesion of the nanoparticles to the bacterial surface and may exhibit their own antibacterial activity, showing a synergistic effect with silver, resulting in lower MIC and MBC values [63].

Accordingly, the differences in MIC and MBC values observed in comparison with those reported in the literature may be due to the coating formed during the synthesis process, since Wypij et al. (2021) used the SF23 strain of *Actinobacteria* to synthesize AgNPs, while Khojasteh-Taheri et al. (2023) used *Caccinia macranthera* extract [59,60]. The BioAgNP from GRAL Bioativos was synthesized using catuaba extract (*Trichilia catigua*), which has antibacterial activity, as demonstrated by Gonçalves Paschoal et al. (2020) [64]. Therefore, considering the synthesis process, it is possible that the coating of the BioAgNPs contains molecules with antibacterial activity, which may act synergistically with silver, resulting in the excellent antibacterial activity of the BioAgNPs. The study of Kimura et al. (2025), who also employed BioAgNPs synthesized from catuaba extract, reported low MICs for *P. aeruginosa* ATCC 27853 of 5.3 µg/mL, a value compatible with that presented in Table 1 [65].

Once the antibacterial activity of BioAgNPs against standard strains had been established, a broth microdilution assay was performed using the multidrug-resistant *A. baumannii*. The isolates chosen for this work were 183, 289, 791, 846, and 860, due to their resistance profiles. All five isolates showed resistance to more than three of the tested antimicrobials, including polymyxin B and colistin, which are considered last-line antibiotics for the treatment of multidrug-resistant *A. baumannii* infections. These isolates were classified as multidrug-resistant [66]. In particular, isolate 846 is considered pan-resistant, that is, it showed resistance to all tested antimicrobials.

The MIC values of BioAgNP for multidrug-resistant *A. baumannii* isolates were close to those obtained for *A. baumannii* ATCC 19606, with no significant difference. This demonstrates that BioAgNPs have potential for combating multidrug-resistant strains. The bacterium *A. baumannii* is responsible for causing hospital-acquired infections and can exhibit resistance to several classes of antibiotics, including last-line antibiotics such as colistin, being a global health problem recognized by the World Health Organization as one of the most critical pathogens [67,68]. This bacterium has a repertoire of virulence factors that make infections lethal and allow survival in various situations [67]. In this context, several studies suggest that AgNPs are a promising alternative for combating multidrug-resistant *A. baumannii*, which is strongly supported by the results presented in Table 2 [61,67,69].

However, an increase in the MBC of the multidrug-resistant isolates was observed when compared to the MBC of *A. baumannii* ATCC 19606, possibly associated with the resistance mechanisms of the isolates. For example, resistance to β-lactams, including carbapenems, fluoroquinolones, macrolides, polymyxins, tetracyclines, especially tigecycline, occurs due to the presence of efflux pumps of the RND family, such as AdeABC, AdeFGH, and AdeIJK [70]. It is possible that the same pumps, overexpressed in resistant strains, are capable of effluxing nanoparticles, which may be responsible for the increase in the MBC of BioAgNPs [71].

The lower quantity of porins hinders the entry of BioAgNPs, decreasing their antibacterial effect. Even so, the MICs and MBCs of BioAgNPs for the five multidrug-resistant isolates are considered low, reinforcing the potential application of BioAgNPs in combating multidrug-resistant bacteria.

### 4.3. Cytotoxicity Assay

AL did not exhibit cytotoxic activity at any of the concentrations tested, indicating that it is a highly safe and biocompatible compound. The result is consistent with that observed by Ahmadian et al. (2021) [72], who concluded that AL is a non-toxic compound capable of inducing cell proliferation and increasing cell viability. Due to the absence of toxicity of AL, it was not possible to calculate the cytotoxic concentration capable of killing 50% of the cells (CC50).

Similarly to AL, HA did not exhibit cytotoxic activity, making it impossible to calculate the CC50. Ahmadian et al. (2021) also observed that HA does not exhibit cytotoxic activity, since it is a natural component of the extracellular matrix (ECM) of tissues, exhibiting low immunogenicity and high biocompatibility [72]. Interestingly, an increase in cell viability was observed at some HA concentrations, especially at 8 mg/mL, indicating that the compound aids in cell proliferation or induces an increase in the metabolic activity of cells. Therefore, neither wound-healing agent exhibited cytotoxic activity, and they were considered safe for use in the formulation.

Unlike AL and HA, BioAgNPs exhibited cytotoxic effects at the highest concentrations tested. From the trend line equation of the scatter plot, the CC_50_ value was calculated, resulting in 132.071 μM or 14,239 μg/mL.

The cytotoxic concentrations of BioAgNPs were relatively low, particularly when compared to the MIC values obtained. The result was consistent with the findings of Tripathi and Goshist (2020), who reported that biogenic AgNPs did not show a cytotoxic effect at concentrations up to 25 µg/mL [73].

The cytotoxic activity of AgNPs is already well documented and is attributed to the fact that their antimicrobial mechanisms of action are not exclusive to bacterial cells, being able to also affect eukaryotic cells. For instance, AgNPs are capable of adhering to biological tissues in a manner similar to the adhesion observed on bacterial surfaces [74].

Once inside eukaryotic cells, AgNPs produce reactive oxygen species (ROS), which induce oxidative stress, leading to apoptosis. AgNPs inhibit the activity of glutathione-synthesizing enzymes, reducing intracellular levels of this important antioxidant. The excess ROS activates apoptosis-related signaling pathways, such as p53, protein kinase B (AKT), and mitogen-activated protein kinase (MAPK), resulting in the cytotoxic effect [75].

BioAgNPs exhibited low cytotoxic activity, possibly related to their particle size, since smaller nanoparticles tend to induce higher toxicity due to increased ROS production [75]. This CC50 value of 14.239 µg/mL is considered safe for topical use, being below 25 µg/mL [76]. Thus, BioAgNP showed an acceptable SI for almost all bacteria tested, except for *S. aureus*, which presented the lowest selectivity index.

### 4.4. Scratch Assay

Cell migration and proliferation of fibroblasts are important processes for burn healing. In the proliferation phase, fibroblasts at the edges of the wound migrate to deeper portions, where they deposit ECM to initiate the formation of new connective tissue. ECM provides support for fibroblasts to migrate and proliferate in the burn, promoting healing and scarring [1,77].

Colangelo et al. (2022) observed increased cell proliferation and migration in treatment containing HA, resulting in significantly faster wound closure than in the control group [78].

These results indicate that HA facilitates cell migration, as it is a natural component of granulation tissue during the healing process, providing a matrix that facilitates cell movement [78]. Hyaluronic acid binds to and activates the CD44 surface receptor, expressed in fibroblasts and keratinocytes, promoting the activation of effectors such as Rac1, RhoA, and ERK, resulting in the reorganization of the cytoskeleton, allowing the cell to migrate [79].

AL also accelerated the wound closure process, likely due to its ability to promote granulation tissue formation and stimulate cell proliferation and migration [80]. AL stimulates the activity of the enzymatic antioxidant system, which promotes the conversion of ROS into hydrogen peroxide. Hydrogen peroxide acts as a second messenger for several growth factors, increasing the tyrosine kinase activity of various proteins [81].

AL at a concentration of 200 µg/mL resulted in lower wound closure compared to 100 µg/mL. This effect may be due to increased hydrogen peroxide production, which can also activate the NF-κB transcription factor. NF-κB is associated with the expression of pro-inflammatory factors, which can interfere with cell regeneration pathways [81]. These findings indicate that HA at 8 mg/mL and LA at 100 µg/mL are promising candidates for wound-healing formulations.

### 4.5. Binary Association of BioAgNPs with HA and AL

A possible explanation for the increased MIC for *A. baumannii* when BioAgNP is associated with HA is the presence of negatively charged functional groups in HA, such as hydroxyl, carboxyl and acetamide. Due to its negative charges, these groups can bind to silver ions, which have positive charges, decreasing the antibacterial effect [82]. In addition, AgNPs exhibit better antibacterial activity at basic pH than at acidic pH, so HA can interfere with the action of BioAgNPs due to its acidic nature [83]. The combined effect of silver ion sequestration by HA and capsule-mediated protection may account for the reduced antibacterial activity observed.

The study by Dresler et al. (2019) demonstrated that AL in plants attenuates toxicity caused by cadmium, a positively charged ion, due to its antioxidant action [84]. One of the proposed mechanisms of antibacterial action of AgNPs is the production of reactive oxygen species, so it is possible that the antioxidant activity of AL also attenuates the antibacterial action of BioAgNPs [53].

The highest value observed was 125 µM or 13,483 µg/mL, which remains lower than the CC_50_ of BioAgNPs (132.071 µM or 14.239 µg/mL). At this concentration, the BioAgNPs exhibit low cytotoxicity toward human cells while maintaining antibacterial activity against the tested pathogens. Moreover, the MIC values in combination remain below 25 µg/mL, which is considered the safety threshold for topical application of silver nanoparticles [76].

### 4.6. Formulation Characterization

The hydrogel formulation was based on hydroxyethylcellulose (HEC), a water-soluble polymer derived from cellulose, widely used as a stabilizer, thickener, or coating agent in pharmaceutical and cosmetic formulations due to its biocompatibility and low toxicity [85].

Glycerin was incorporated into the formulation as an emollient, promoting epidermal softness and flexibility, and as a humectant, maintaining skin hydration and moisture [86]. Finally, a paraben-free preservative system composed of methylisothiazolinone and phenoxyethanol was added. This preservative keeps the formulation preserved for a period of 6 to 12 months, depending on the conditions of use and storage, having an effect against fungi, Gram-positive and Gram-negative bacteria [87].

BioAgNPs were added at a concentration of 125 µM or 13.483 µg/mL, based on the result of the association assay. HA and AL were added at 8 mg/mL and 100 µg/mL, which exhibited the most favorable outcomes in the Scratch assay.

Both centrifugation and preliminary stability tests demonstrated that the formulation was able to maintain physical integrity without phase separation or precipitation, although the high temperature used in the preliminary stability test resulted in a slight color change.

The relative density of both FAC and BC showed a slight increase after the preliminary stability test; however, the analysis did not reveal a statistically significant difference between FAC before and after the test (*p* = 0.46), nor between BC before and after preliminary stability (*p* = 0.42).

Viscosity is inversely related to spreadability [88]. Increased viscosity is associated with greater formulation stability, indicating adequate interaction among the components. Moreover, more viscous formulations exhibit reduced flow behavior, which favors the prolonged retention at the application site. This characteristic is particularly advantageous in formulations intended for topical wound treatment, as it enhances coverage and protection of the injured area, contributing to microenvironment favorable for tissue repair. The higher viscosity of the FAC prolongs the contact time at the application site and the delivery rate of the active ingredients [88,89]. Sustained delivery of the bioactive compounds may allow for reduced application frequency, potentially enabling once-daily administration instead of multiple daily doses [90].

In Figure 8, it is possible to observe small peaks related to the presence of silver. The silver concentration was intentionally kept low to remain within the established cytotoxic safety window determined by MTT assays. At this concentration range, quantitative surface-sensitive techniques such as EDX may not provide strong elemental signals due to detection limits, as shown in Figure 8. However, the antibacterial activity observed confirms the functional presence of BioAgNPs within the hydrogel matrix. Importantly, the MIC values remained below the cytotoxic concentration, supporting that the silver content incorporated into the hydrogel was sufficient to exert antibacterial activity while maintaining biocompatibility. The high signals corresponding to carbon (C) and oxygen (O) are attributed to the molecular composition of AL, HA, hydroxyethylcellulose, and glycerin, which are rich in these elements, while sodium (Na) is a component of Disodium EDTA.

### 4.7. Antibacterial Activity of the Formulation

#### 4.7.1. Agar Drop-Diffusion Assay

In Figure 9, no inhibition halo was observed around the formulation, possibly due to the low diffusivity of silver in agar. The study of Cornette de Saint Cyr et al. (2021) demonstrated that the diffusivity of silver in agar is 2.7 × 10^−9^ m^2^/s and that a minimum concentration of 20 µg/mL is necessary to observe the action of silver on bacterial growth within 24 h [91]. The concentration of silver in FA is 13.483 µg/mL, which is below the minimum concentration required to produce a visible inhibition halo. Nevertheless, it was observed that there was no bacterial growth at the site where the formulation was inoculated for the bacteria *A. baumannii* and *P. aeruginosa*. In contrast, *S. aureus* showed bacterial growth, although with partial inhibition. For all three bacterial strains, the base formulation did not exhibit antibacterial activity.

#### 4.7.2. Antimicrobial Efficacy Test

According to the Brazilian Pharmacopoeia, the approval criteria require a reduction of at least two logarithmic units in bacterial count after 14 days, with no subsequent increase up to 28 days. However, in the graphs presented in Figure 10, bacterial growth was entirely suppressed with a decrease of approximately eight logs, thus exceeding the required criteria for all three bacteria.

The results of the multidrug-resistant *A. baumannii* isolates were similar to the reference strains, showing complete elimination from the FA within 2 h of contact. An interesting behavior was observed for isolate 860. Unlike the other strains, this isolate was unable to survive in the base formulation for up to 12 h, showing complete elimination after 4 h even in the absence of antibacterial agents, a result consistently observed across replicates. This may be due to intrinsic strain factors that lead to low survival. Recent studies suggest that genetic or phenotypic alterations may compromise the survival of specific strains, requiring further testing, such as genomic profiling or analysis of surface protein expression, to elucidate this behavior [92].

Therefore, the antimicrobial efficacy assay complemented the drop-diffusion test, as it evaluates bacterial elimination under direct contact conditions, which more closely resemble topical application. The rapid antibacterial activity of FA, capable of eliminating bacteria within 2 h of contact, is particularly advantageous, since rapid topical application at the beginning of the wound treatment can control planktonic bacteria, preventing biofilm formation [93].

#### 4.7.3. Time–Kill Curve Assay

The presence of culture medium favored the bacterial growth of *S. aureus*, which was already more resistant to the antibacterial action of BioAgNPs compared to the other bacteria, allowing its continuous growth. However, FA reduced bacterial counts by approximately one log unit, which, although not sufficient to be considered a bactericidal action, still indicates antibacterial activity.

Again, the survival of *S. aureus* following the treatment with FA may be attributed to the cell wall structure of Gram-positive bacteria. Accumulation in the outer membrane is one of the key antibacterial mechanisms of AgNPs in Gram-negative bacteria, where this structure plays a crucial role in cellular integrity. In Gram-positive bacteria, the absence of an outer membrane, which allows silver to pass through porins and favors the adhesion of AgNPs to this structure, limits the entry of BioAgNPs into the bacterial cell [53].

In the study by Ferreira et al. (2023), the time–kill curves of *S. aureus* demonstrated a significant inhibition in bacterial growth [94]. However, the AgNP concentration used was 30 µg/mL, nearly twice that present in FA (13,483 µg/mL). Thus, increasing the BioAgNP concentration may enhance the reduction of *S. aureus* by up to two log units, although such adjustments must remain within established safety limits for topical application.

The exponential decrease observed in the curves of *P. aeruginosa* ATCC 27853, *A. baumannii* ATCC 19606, and isolate 846 (Figure 12a,b,d) when treated with FA may be due to damage to the cell membrane, as demonstrated by Singh et al. (2021), who observed pores in the membrane, cell lysis, and leakage of intracellular contents, using scanning electron microscopy [95].

Chen et al. (2019) reported a significant decrease in the growth of multidrug-resistant *A. baumannii* when treated with AgNP at 20 µg/mL, with a two-log decrease after about 18 h [96]. These findings further support that AgNPs exhibit antibacterial activity against multidrug-resistant strains of *A. baumannii*. The effectiveness of FA against the pan-resistant isolate 846 reinforces its potential as a therapeutic candidate in clinical settings facing outbreaks of multidrug-resistant bacteria, such as intensive care units and burn centers.

The complete elimination of bacteria in nutrient-enriched medium confirms that the antibacterial activity of FA can be effective in clinical scenarios with abundant exudate and injured tissue, which provide nutrients for pathogens, in the face of the growing scenario of clinical antimicrobial resistance.

## 5. Conclusions

The hydrogel formulation containing biogenic silver nanoparticles, hyaluronic acid, and allantoin represents an innovative and effective therapeutic strategy for burn treatment, combining antibacterial activity with wound-healing promotion.

The antibacterial activity of BioAgNPs was confirmed by MIC determination, whereas HA and AL did not exhibit action against bacteria at the tested concentrations. All three active ingredients presented low cytotoxicity, particularly HA and AL, allowing the determination of safe concentrations for BioAgNPs use. HA and AL promoted wound closure by stimulating cell proliferation and migration in the Scratch assay, highlighting their regenerative potential. Although the combination of HA and AL slightly increased the MIC of BioAgNPs, the values remained below the cytotoxic threshold, ensuring formulation safety and low toxicity.

Microbiological evaluation of the hydrogel revealed excellent antibacterial activity, primarily against *Pseudomonas aeruginosa* and *Acinetobacter baumannii*, two major pathogens in burn infections. Notably, the formulation was also effective against multidrug-resistant *A. baumannii* isolates, indicating its potential as an alternative strategy for controlling resistant infections. Time–kill curves indicated a rapid antibacterial effect, with significant elimination of pathogens within 2 h, which constitutes an important clinical differential in the prevention of biofilm formation and systemic complications such as sepsis.

Although the activity against *Staphylococcus aureus* was insufficient to achieve a bactericidal effect against Gram-positive bacteria, these findings suggest opportunities for formulation optimization, such as increasing the BioAgNP concentration, as long as it remains below the safe concentration for use, or incorporating antimicrobial agents with action directed against Gram-positive bacteria.

Overall, the developed hydrogel combining BioAgNPs, HA, and AL demonstrated antibacterial activity against MDR bacteria and promoted fibroblast migration in vitro, indicating potential for topical treatment of infected burn wounds. Future studies should explore formulation optimization, synergistic antimicrobial combinations, and in vivo evaluation in burn injury models.

## Figures and Tables

**Table 1:** Minimum inhibitory concentration (MIC) and minimum bactericidal concentration (MBC) values of biogenic silver nanoparticles (BioAgNP) determined from the broth microdilution assay.

	MIC (μM)	MBC (μM)	MIC (µg/mL)	MBC (µg/mL)	MBC/MIC
AB ATCC 19606	0.976–1.953	15.625–31.25	0.105–0.211	1.685–3.371	16
PA ATCC 25853	7.812–15.625	31.25–62.5	0.843–1.685	3.371–6.742	4
SA ATCC 6538	31.25–62.5	500–1000	3.371–6.742	53.634–107.868	16

AB: *Acinetobacter baumannii*; PA: *Pseudomonas aeruginosa*; SA: *Staphylococcus aureus*; MIC: minimum inhibitory concentration; MBC: minimum bactericidal concentration.

**Table 2:** Minimum inhibitory concentration (MIC) and minimum bactericidal concentration (MBC) values of biogenic silver nanoparticles (BioAgNP) for multidrug-resistant clinical isolates of *Acinetobacter baumannii*.

Isolates	MIC (μM)	MBC (μM)	MIC (µg/mL)	MBC (µg/mL)	MBC/MIC
AB183	1.953–3.906	250–500	0.211–0.421	26.967–53.934	128
AB289	0.976–1.953	31.25–62.50	0.105–0.211	3.371–6.742	32
AB791	1.953–3.906	125–250	0.211–0.421	13.483–26.967	64
AB846	1.953–3.906	125–250	0.211–0.421	13.483–26.967	64
AB860	1.953–3.906	62.50–125	0.211–0.421	6.742–13.483	32

AB: *Acinetobacter baumannii*; MIC: minimum inhibitory concentration; MBC: minimum bactericidal concentration.

**Table 3:** Selectivity index of biogenic silver nanoparticles (BioAgNP) for standard strains of *Acinetobacter baumannii*, *Staphylococcus aureus*, and *Pseudomonas aeruginosa*, and for multidrug-resistant isolates of *Acinetobacter baumannii*.

Bacteria	Selectivity Index
*S. aureus* ATCC 6538	3.895
*P. aeruginosa* ATCC 27853	15.579
*A. baumannii* ATCC 19606	124.644
*A. baumannii* 183	63.322
*A. baumannii* 289	124.644
*A. baumannii* 791	63.322
*A. baumannii* 846	63.322
*A. baumannii* 860	63.322

**Table 4:** Minimum inhibitory concentrations (MIC) of biogenic silver nanoparticles (BioAgNP) in association with hyaluronic acid (HA) at 8 mg/mL and allantoin (AL) at 100 μg/mL.

	MIC BioAgNP Combined with HA at 8 mg/mL (μM)	MIC BioAgNP Combined with AL at 100 μg/mL (μM)
*A. baumannii* ATCC 19606	7.81–15.62	3.90–7.81
*P. aeruginosa* ATCC 27853	7.81–15.62	7.81–15.62
*S. aureus* ATCC 6538	15.62–31.25	62.5–125.00

BioAgNP: biogenic silver nanoparticles; HA: hyaluronic acid; AL: allantoin.

**Table 5:** Relative density of the formulation with active ingredients and preservative (FAC) and of the base without active ingredients and with preservative (BC) before the preliminary stability test.

		Relative Density (g/mL)
Before the preliminary stability test	BC	1.00994 ± 0.0025
	FAC	1.02087 ± 0.0025
After the preliminary stability test	BC	1.01557 ± 0.0789
	FAC	1.02286 ± 0.00287

BC: formulation base without active ingredients and with preservative; FAC: formulation with active ingredients and preservative.

## Data Availability

The original contributions presented in this study are included in the article. Further inquiries can be directed to the corresponding author.

## Abbreviations

The following abbreviations are used in this manuscript:

AB	*Acinetobacter baumannii*
AL	Allantoin
ATCC	American Type Culture Collection
BC	Base without active ingredient containing preservative
BHI	Brain heart infusion
BioAgNP	Biogenic silver nanoparticles
CC_50_	Cytotoxic concentration
CFU	Colony-forming units
CLSI	Clinical and Laboratory Standards Institute
DLS	Dynamic light scattering
DMEM	Dulbecco’s Modified Eagle Medium
EDTA	Ethylenediamine tetra-acetic acid
FA	Active formulation without preservative
FAC	Active formulation containing preservative
FBS	Fetal bovine serum
HA	Hyaluronic acid
ISO	International Organization for Standardization
MBC	Minimum bactericidal concentration
MH	Mueller Hinton
MIC	Minimum inhibitory concentration
MTT	3-(4,5-dimethylthiazol-2-yl)-2,5-diphenyltetrazolium bromide
NCCLS	National Committee for Clinical Laboratory Standards
PA	*Pseudomonas aeruginosa*
PBS	Phosphate-buffered saline
PDI	Polydispersity index
SA	*Staphylococcus aureus*
SI	Selectivity index
TEM	Transmission electron microscopy
UEL	State University of Londrina
UEM	State University of Maringá
XDR	X-ray diffraction spectroscopy
